# Distinguishing Male and Female Chinese Rose Beetles, *Adoretus sinicus*, with an Overview of *Adoretus* Species of Biosecurity Concern

**DOI:** 10.1673/031.011.6401

**Published:** 2011-05-20

**Authors:** Grant T. McQuate, Mary Liz Jameson

**Affiliations:** ^1^ USDA-ARS, U.S. Pacific Basin Agricultural Research Center (PBARC); ^2^ Department of Biological Sciences, Wichita State University, 537 Hubbard Hall, Wichita, Kansas 67260

**Keywords:** Scarabaeidae, invasive species

## Abstract

The Chinese rose beetle, *Adoretus sinicus* Burmeister (Coleoptera: Scarabaeidae: Rutelinae: Adoretini), is a broadly polyphagous scarab beetle that is economically important and causes damage to a wide variety of host plants including agricultural crops and ornamentals in Southeast Asia, China, the Hawaiian Islands and several other Pacific Islands. The species has become established in numerous regions and is of biosecurity concern because importation of this species to other regions poses a threat to agriculture due to its generalist herbivore feeding habits. Field and laboratory research directed towards control of the species is hampered by the lack of characteristics that allow accurate determination of the sexes on live beetles in the field. Here, three recognizable and reliable non-destructive morphological differences between the sexes of *A. sinicus* are documented: (1) the form of the terminal sternite; (2) the length to width ratio of protarsomere 1, and; 3) the ratio of the combined length of protarsomeres 2–4 to the length of protarsomere 1. Because many *Adoretus* species are of biosecurity concern, and because tools to identify *Adoretus* species are lacking, we review the natural history and research on control associated with *A. sinicus* as well as the genus as a whole.

## Introduction

**Figures 1–2.**
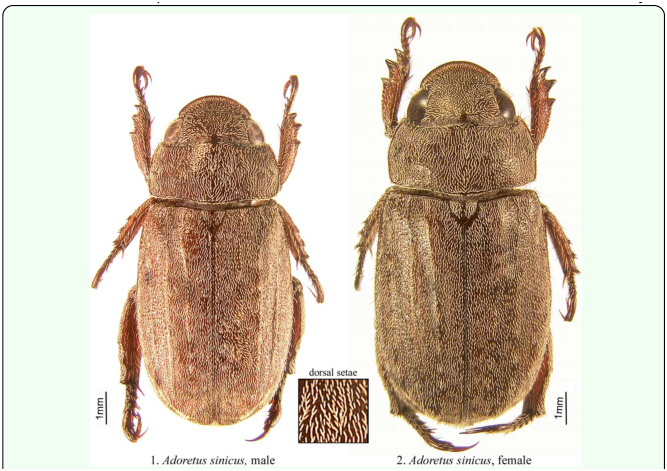
Habitus of *Adoretus sinicus:* 1) Male. 2) Female. Inset shows close-up of dorsal setae. High quality figures are available online.

In the Hawaiian Islands, the introduction of non-native animals and plants has caused millions of dollars in crop losses, extinction of native species, destruction of native forests, and in excess of $40.8 million in government-sponsored spending in 2006 for biosecurity initiatives (Hawaii Department of Land and Natural Resource 2008). Well over 2,500 arthropod species have been introduced to the Hawaiian Islands with continued establishment rates at an alarming ten to twenty species per year (Asquith 1995). One non-native species in the Hawaiian Islands is the Chinese rose beetle, *Adoretus sinicus* Burmeister (Coleoptera: Scarabaeidae: Rutelinae: Adoretini) (Figures 1–2). The species is native to Japan and Taiwan (Mau and Kessing 1991), but its distribution now also includes China (including Hong Kong), Indonesia, Cambodia, Singapore, Thailand, Vietnam, the Marianas Islands, the Caroline Islands and the Hawaiian Islands (CAB International 1981). In the Hawaiian Islands, it was first reported on Oahu in 1891 (Riley and Howard 1893). By 1893, the species was “rapidly becoming a most serious pest” (Riley and Howard 1893). Initial identification provided the name *Adoretus umbrosus* L.; later, it was referred to as *A. tenuimaculatus* Waterhouse (Pemberton 1964). Nineteen years after its noted pest status, the German ruteline systematist Friedrich Ohaus correctly identified the beetle as *A. sinicus.*

*Adoretus sinicus* probably came to Hawaii in the larval stage in soil with imported plants from China, Taiwan, Java, or Timor (Ohaus 1935; Pemberton 1964). By 1898 it was established on all major Hawaiian Islands (Koebele 1898), and in 1917 it was referred to as “one of the worst garden pests of the Islands” (Muir 1917). In areas where it has been introduced, adults can cause heavy damage to crops and ornamental plants. Despite much research devoted to control of the species (including pesticides, parasitoids, pathogens, and pheromones) control has been largely ineffective (Beardsley 1993). To date, *A. sinicus* remains an abundant pest throughout the Hawaiian Islands (e.g., Beardsley 1993; Furutani et al. 1995; Siemann and Rogers 2003) and is considered a threat to the west coast of the United States (Ritcher 1948). The species is included on the USDA-APHIS Regulated Plant Pest List (USDA-APHIS 2000) and has been detected on floors of empty sea cargo containers in storage in Australia (Stanaway et al. 2001). Fortunately, biosecurity protocols have become more efficient in recent years, and *A. sinicus* has not become established in the mainland United States. Nevertheless, *A. sinicus* is considered a species with moderate potential for invasive risk in the United States based on factors that include: 1) areas with potentially suitable climate exist; 2) entry potential into a new country based on historical data and interceptions; 3) potential economic impact if the species were to become established; and 4) specific hosts that the species can damage (Gregory et al. 2005). Because it has become established in numerous regions, it is clearly a species of concern for biosecurity.

Methods of control are needed for the Chinese rose beetle, both to minimize damage caused to agricultural and ornamental crops in Hawaii as well as to reduce the risk of its introduction to the U.S. mainland. The lack of reliable, non-destructive methods for sex determination impedes field and laboratory research. Determination of males and females in most species of *Adoretus* is problematic. Unlike many genera of leaf chafers (Rutelinae: Scarabaeidae), there is amazingly little external sexual dimorphism. Within the Rutelinae, standard sexual determination can be made based on a variety of characters, including differences in the form of the protarsal claws, protarsi, metatibial apex, metatibia, metacoxa, metafemur, metatrochanter, and abdominal sternites. However, as stated by Baraud (1985), most *Adoretus* species lack noticeable sexual dimorphism. Currently, determination of sexes in adult *A. sinicus* requires killing and dissection of specimens (e.g., Hession et al. 1994). Our research sought more readily accessible, external morphological characters that would not necessitate destruction of live specimens. In support of further research to improve control of the Chinese rose beetle, an assessment of characteristics that can be used for non-destructive differentiation of the sexes is provided. Additionally, because background literature on Chinese rose beetle is scattered among a number of publications, many of which are not readily available, a review of the basic biology of this species and past efforts of control is also included. The assessment provided here of characteristics useful for differentiation of sexes in the Chinese rose beetle may also be helpful in sex determination of other species of *Adoretus*, including known pests that are also of biosecurity concern because of the potential for invasion through the movement of potted plants across geographic regions. In recognition of the potential agricultural and biosecurity threat of *Adoretus* species, we also review closely related and economically important *Adoretus* species.

## Biology of the Chinese Rose Beetle

The Chinese rose beetle is a generalist foliage feeder and is primarily active at night (Ebesu 2003). Damage to ornamental plants and crops is caused by interveinal feeding that creates a lace-like appearance and greatly reduces photosynthetic capabilities of the plant (Furutani et al. 1995). The adult Chinese rose beetle has been reported to feed on over 250 species and approximately 56 families of plants (Habeck 1964) or on over 500 plant species (Hession et al. 1994). Host plants include many economically important plants such as broccoli (*Brassica oleracea* var. *italica* Plenck), cabbage (*Brassica oleracea* var. *capitata* L.), cacao (*Theobroma cacao* L.), Chinese broccoli (*Brassica oleracea* L. var. *alboglabra*), Chinese cabbage (*Brassica rapa* L. subsp. *chinensis* [L.] Hanelt), chiso (*Perilla frutescens* [L.] Britton), corn (*Zea mays* L.), cotton (*Gossypium barbadense* L.), cucumber (*Cucumis sativus* L.), eggplant (*Solanum melongena* L.), ginger (*Zingiber officinale* Roscoe), grape (*Vitis labrusca* Bailey), green beans (*Phaseolus vulgaris* L.), jack fruit (*Artocarpus heterophyllus* Lam.), okra (*Hibiscus esculentus* L.), peanuts (*Arachis hypogaea* L.), Oriental persimmon (*Diospyros kaki* Thunb.), raspberry (*Rubus niveus* Thunb.), roses (*Rosa* spp.), salak palm (*Salacca zalacca* Gaerther), soybean (*Glycine max* L.), star fruit (*Averrhoa carambola* L.), strawberry (*Fragaria chiloensis* [L.] Duch.), sweet potato (*Ipomoea batatus* [L.]), taro (*Colocasia esculenta* [L.] Schott) and tea (*Camellia sinensis* L.) (Mau and Kessing 1991; Arita et al. 1993; Zee et al. 2003).

Adults emerge at dusk and peak feeding and mating occurs about 30 minutes after sunset (Tsutsumi et al. 1993). As is common with many other nocturnal species of scarab beetles, adults are readily monitored using light traps. Sex ratio in the field is approximately 1:1, although about 27.9% of adults collected at light traps are males (Habeck 1964). During the day, adults are found under leaves, loose bark, or are shallowly buried in the soil (Williams 1931). Adults preferentially feed on leaves and plant species that are relatively high in nonstructural carbohydrates (Arita et al. 1993) and prefer leaves with feeding or other types of damage (Pemberton 1959). Plants produce increased levels of ethylene gas both after ethephon treatment and after leaves are damaged by insect feeding. It appears that the high levels of ethylene gas emission “are involved in the formation of a plant volatile(s) that serves as an attractant and feeding stimulus for the Chinese rose beetle” (Arita et al. 1988). Such an attractant, however, has not yet been identified.

Developmental rate of *A. sinicus* from egg to adult varies greatly depending on temperature and quality of larval substrate. Development from egg to adult averages about 15 weeks in the laboratory (Habeck 1964) or 6–7 weeks in the field (Mau and Kessing 1991). Oviposition begins seven days after pairing of mature males and females (Habeck 1964). Females oviposit in the soil about 4 cm below the surface. Based on 6 field-collected females, females lay 22–89 eggs each (average = 54). Eggs are oval (∼1.5 × 1.0 mm) and white (Habeck 1963). The egg stage may last approximately 12–16 days at 24.0°C or 7–13 days at 28.6°C (Habeck 1964). The larval stage includes three instars. Duration of the instars averages 19.6–22.8, 14.5–16.8, and 34.3–44.4 days, respectively (Habeck 1964). Larvae are c-shaped, white grubs that live in rich soil, leaf litter, decaying vegetation, or compost, where they feed on dead plant tissues (Mau and Kessing 1991). They apparently do not attack living vegetable tissues (Mau and Kessing 1991; Williams 1931). The pupa, which is approximately 6.0– 12.0 mm, is covered with dense, short setae. The pupal stage lasts 11–17 days, averaging about 14 days (Habeck 1964). The adult beetle (Figures 1, 2) is oblong oval, reddish-brown and covered with creamy, dense, scale-like setae, giving the beetle an overall grayish appearance. Body length varies from ∼10–12 mm in length. Species identification for *A. sinicus* is based on form of the male genitalia (Figure 10). Field-collected adults have been found to live for as long as eight weeks in the laboratory (Habeck 1964).

The characteristic interveinal defoliation pattern of the Chinese rose beetle is caused by the beetle's unusual mouthparts. The labrum is produced ventrally at the middle and forms a tooth-like process. This process completely separates the mandibles and maxillae into two independent chewing apparati that are incapable of meeting in the middle. When feeding on a leaf surface, the beetle uses only one side of its mouth at a time, thus producing paired holes in leaves and often leaving a narrow strip of leaf intact in the middle (Arrow 1917).

Because adults cause heavy damage to crops and ornamental plants, research has been devoted to control of the species. Despite numerous attempts using many methods, control of the Chinese rose beetle has proved to be problematical. Tests to develop effective control techniques have included the use of parasitoids, pathogens, pesticides, food lures, and aggregation pheromones.

Parasitic Hymenoptera from the families Scoliidae (*Campsomeris marginella modesta* Smith) and Tiphiidae (*Tiphia segregata* Crawford) were introduced to Hawaii from the Philippines to control the introduced scarab beetles *A. sinicus* and *Anomala orientalis* (Waterhouse) (Scarabaeidae: Rutelinae: Anomalini) (Muir 1917, 1919). Over 14,000 eggs, pupae, and adults of *C. marginella modesta* were imported to Hawaii between 1915–1917. In the short term, there were immediate declines in the target scarabs. Prior to importation of the wasp, over 3,500 *Anomala* grubs were found in 1/20th of an acre; only four *Anomala* grubs were found in the same area in 1919 (Muir 1919). Muir (1919) hypothesized that biocontrol success would lead to “extinction” of *A. orientalis* in the region, and that *C. marginella* would maintain itself on grubs of the Chinese rose beetle. In the long term, however, control by these parasitoids has been only slightly effective (Pemberton 1964). In another attempt, importation of 24,000 *Adoretus* grubs from the Philippines that were parasitized with *Tiphia lucida* Crawford also failed (Pemberton 1964).

Treatments using the entomopathogenic fungi *Metarhizium* and *Beauveria* have been conducted on the Chinese rose beetle. Success of using the green muscardine fungus, *Metarhizium anisopliae* (Metschnikov), against *A. sinicus* are conflicting. Koebele (1897) and Williams (1931) reported some success against the Chinese rose beetle, particularly during the wet season. Three strains of *M. anisopliae* (M346, M2151, and M2162) were tested on larvae of *A. sinicus* with success (Tsutsumi et al. 1993), but effectiveness needs to be replicated due to the high mortality in both controls and experimental treatments. Consistent infection in scarabs may be limited to *Beauveria brongniartii* (Saccardo) Petch or large-spored varieties of *M. anisopliae* var. *majus* (Metch.) Sorokin, and research efforts should be directed towards these fungal strains and species (Jackson and Klein 2006).

Entomopathogenic nematodes in the families Steinernematidae and Heterorhabditidae could be effective control agents for scarab beetle larvae or adults and could minimize the use of pesticides. However, tests on adult *A. sinicus* using the nematodes *Steinernema carpocapsae* (Weiser) and *Heterorhabditis* sp. MB7 (Maui isolate) were ineffective (Hara et al. 1989), and tests on larvae were also ineffective (Tsutsumi et al. 1993).

Broad spectrum organophosphate pesticides, like carbaryl, have been used for Chinese rose beetle control, as they have been used for control of Japanese beetles and other scarab beetles. Because of the potential for significant non-target effects of organophosphate insecticide use, development of effective reduced risk insecticides is needed. An azadirachtin-based pesticide has been tested and found to be promising (Arita Tsutsumi et al. 1995), and imidicloprid-based systemic pesticides have also been found to help in control of Chinese rose beetle. At present, though, pesticide registrations are limited for Chinese rose beetle control in Hawaii, especially considering the wide range of crops that can be attacked by *A. sinicus.*

Food-type lures, as have been developed for the Japanese beetle (*Popillia japonica* Newman) (Scarabaeidae: Rutelinae: Anomalini) (e.g., Ladd and McGovern 1980), could be developed for *Adoretus* species. Alternatively, it may be possible to identify attractive herbivory-induced plant volatiles such as have been found for other scarab beetle species (Loughrin et al. 1995, Reinecke et al. 2002). This possibility is encouraged by the observation that “host plants that were previously fed upon by the beetle were more desirable as a food source than untouched plants of the same species” (Pemberton 1959, Arita et al. 1988). Patterns of feeding preference could be used for management and control of *A. sinicus.* For example, *A. sinicus* prefers recently matured leaves in the uppermost part of the plant, whereas *A. versutus* prefers younger leaves in the lower part of the plant (Tsutsumi et al. 1993).

Pheromones can be used to disrupt behavior, reduce populations, and monitor for outbreaks of scarab pests (Jackson and Klein 2006). Preliminary observations indicated that pheromones may be involved in the attraction of *A. sinicus* and *A. versutus* to mating sites (Tsutsumi et al. 1993), with the probable existence of an *A. sinicus* pheromone sex attractant (Hession et al. 1994). A synthetic sex attractant, produced by females and primarily attractive to males (“Japonilure”), was successfully developed for the Japanese beetle (Tumlinson et al. 1977 1979, Doolittle et al. 1980) and joint presentation of Japonilure and food-type lure provided a lure “of outstanding attractancy” (Ladd et al. 1981). A sex attractant has also been discovered for *Anomala orientalis* (Zhang et al. 1994) and shows promise for population management by mating disruption (e.g., Wenninger and Averill 2006). Promising attractants have been successfully developed for at least one other *Adoretus* species. The synthetic unsaturated terpene alcohols nerolidol (3,7,11-tri-methy 1-1,6,10-dodecatrien-3-ol) and geraniol ([E]3,7,dimethyl-2,6-octodien-1-ol) were shown to be highly attractive to the Australian *A. tessulatus* Burmeister (Donaldson 1986). Both chemicals are found widely in the essential oils of flowers and plants. These results provide encouragement that comparable food-based and sex attractant lures could also be developed for the Chinese rose beetle. However, initial tests of synthetic lures developed for Japanese beetles and other scarab beetles (including geraniol) failed to identify effective attractants for *A. sinicus* and *A. versutus* (Tsutsumi et al. 1993), and preliminary trials of nerolidol also failed to demonstrate attractiveness for *A. sinicus* (GTM, unpublished). Further semiochemical research is clearly needed with *A. sinicus.*

## Materials and Methods

**Insects.** Beetles were collected at night, in the vicinity of Hilo, HI, from four different host plant species (copperleaf, *Acalypha wilkesiana* Muell.-Arg. [Euphorbiaceae]; rose, *Rosa* sp. [Rosaceae]; pilau maile, *Paederia foetida* L. [Rubiaceae]; and wax apple, *Syzygium samarangense* (Blume) Merrill and Perry [Myrtaceae]) from Jan.–Sept., 2009. Collected beetles were held outdoors in an environment that included castor bean (*Ricinus communis* L.); [Euphorbiaceae] plants on which they could feed for at least two weeks before beetles were evaluated. The holding time was selected in order that there would be an opportunity for wearing of the external teeth of the protibia. The external teeth of the protibia tend to be more acute in the male (less acute in the female), but acuteness is subject to wear that rounds the apices of the teeth. Following the two week holding time, beetles were killed by freezing in a So-Low ultra low temperature freezer (So-Low Environmental Equipment, www.so-low.com) maintained at -70° C, and then external morphological characters of potential use in sex determination were evaluated.

**Figures 3–4.**
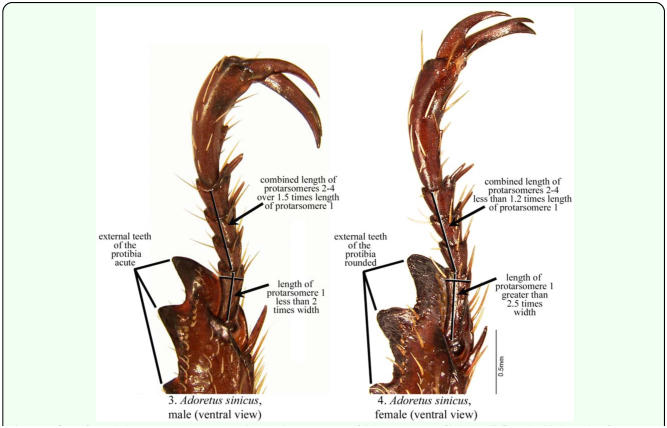
Sexual characteristics associated with the protarsus of *Adoretus sinicus:* 3) Male. 4) Female. High quality figures are available online.

**Figures 5–6.**
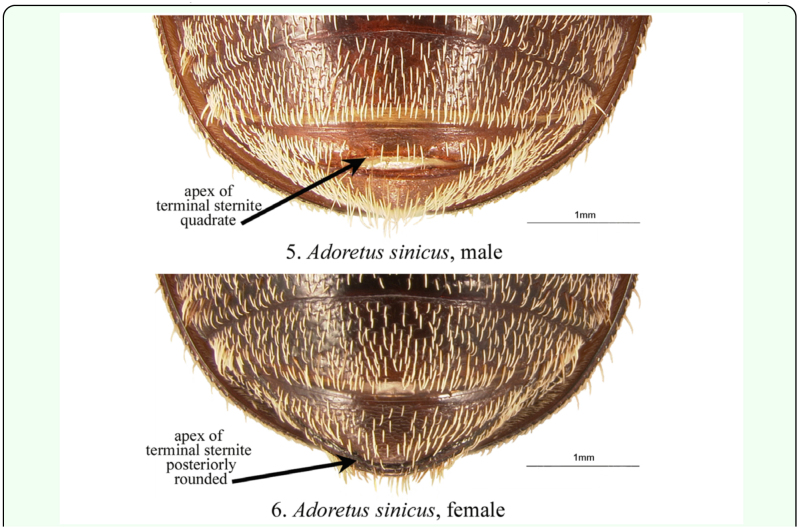
Sexual characteristics associated with the terminal sternite (ventral view) of *Adoretus sinicus:* 5) Male. 6) Female. High quality figures are available online.

The following measurements were made on each individual: total body length (tip of the head to the tip of the closed elytra), maximum body width (maximum width of the elytra, typically at about the middle of the elytra), length of protarsomere 1 (from the top of the condyle to the apex of the protarsomere; see Figures 3, 4), maximum width of protarsomere 1, and the combined length of protarsomeres 2–4 (from the base of protarsomere 2 to the apex of protarsomere 4; see Figures 3, 4). All protarsomere measurements were made using the ventral side of the right front leg. For each specimen, external teeth of the protibia (Figures 3, 4) were assessed as being acute or rounded (Figures 3, 4) and whether the apex of the terminal sternite was quadrate or rounded posteriorly (Figures 5, 6). Each specimen was then dissected for definitive sex identification based on the presence or absence of the male genitalia. Presence or absence of eggs was also noted and, if present, maximum length and width were recorded for two randomly selected eggs. One hundred specimens of each sex were evaluated for the above morphological characters. Observations on the form of the terminal sternite were made with a Leica Wild M3Z dissecting microscope (Leica Microsystems, Inc., www.leicamicrosystems.com) that, in conjunction with a measurement scale etched in a glass slide, was also used to measure body length and body width. Tarsomeres were measured with a Leica Wild M8 microscope with a 1.6X objective adapter and a calibrated ocular micrometer. Digital images of specimens and structures were captured using the Auto-Montage imaging system by Syncroscopy (Synoptics Inc., www.synoptics.co.uk). Images were edited in Adobe Photoshop CS2 (Adobe Systems Inc., www.adobe.com) (background removed, contrast manipulated).

### Statistical Analysis.

Significance of differences by sex in body length and width, protarsomere 1 length, protarsomere width, protarsomere length : width ratio, and protarsomeres 2–4: protarsomere 1 ratio were tested by ANOVA (JMP 2007).

## Results

**Figure 7.**
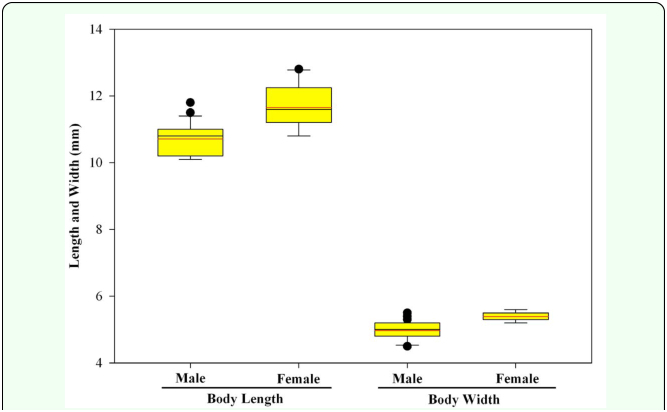
Comparative length and width of male and female adult Chinese rose beetles. Lower and upper boundaries of the boxes are 25^th^ and 75^th^ percentile values. Whiskers below and above the boxes are 10^th^ and 90^th^ percentile values. Black line is median value. Red line is average value. Outlier values are indicated by points outside the whiskers. High quality figures are available online.

**Figure 8.**
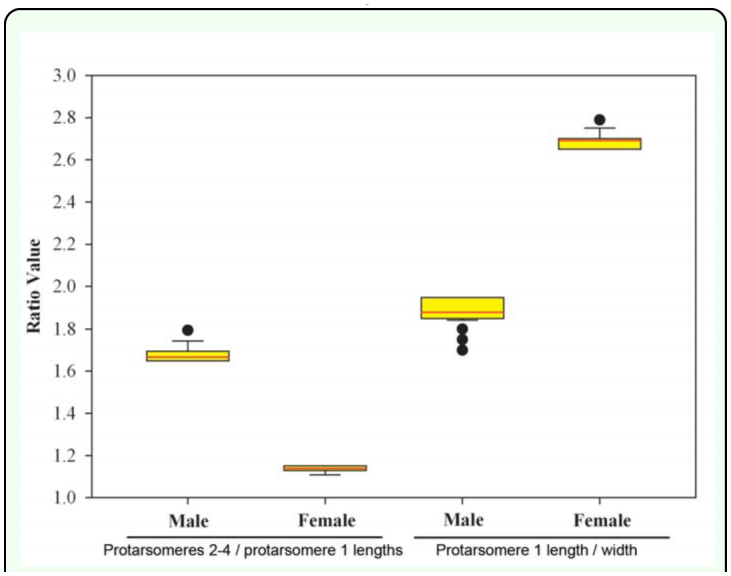
Comparative ratios of lengths of protarsomeres 2–4 combined divided by protarsomere 1 and protarsomere 1 length divided by width for male and female adult Chinese rose beetles. Lower and upper boundaries of the boxes are 25^th^ and 75^th^ percentile values. Whiskers below and above the boxes are 10^th^ and 90^th^ percentile values. Black line is median value. Red line is average value. Outlier values are indicated by points outside the whiskers. High quality figures are available online.

**Figure 9.**
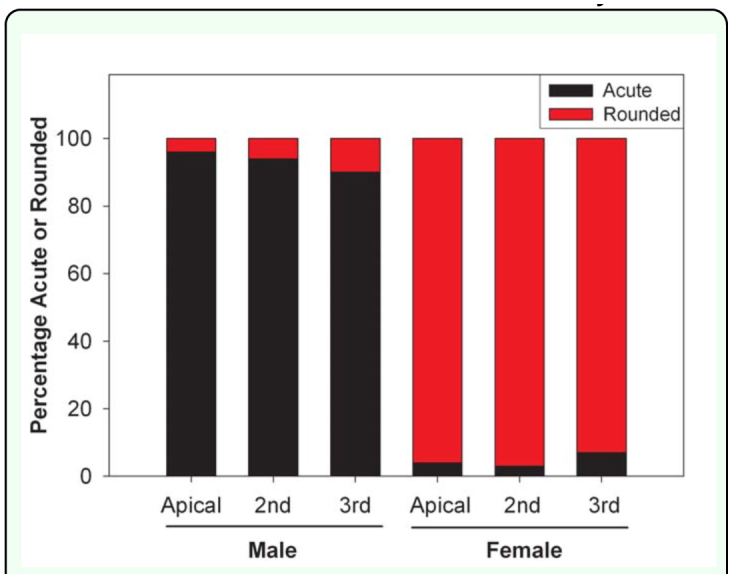
Percentages of observations of the form of the tips of the three external teeth of the protibia noted to be acute versus rounded for male and female adult Chinese rose beetles (based on observations of 100 males and 100 females). High quality figures are available online.

On average, females were significantly larger (based on body length and width) than males (mean ± SE: *length* ♀: 11.65 ± 0.067 mm; ♂: 10.71 ± 0.047 mm; *F* = 130.91, df = 1, 198, *p* < 0.0001; *width* ♀: 5.39 ± 0.014 mm; ♂: 4.97 ± 0.024 mm; *F* = 228.17, df = 1, 198, *p* < 0.0001). However, obvious overlap between the sexes make these measurements unreliable for sex determination (Figure 7). Protarsomere 1 was significantly longer in females (♀: 0.54 ± 0.00070 mm; ♂: 0.37 ± 0.00071 mm; *F* = 29166.35, df = 1, 198, *p* < 0.0001; Figures 3, 4). However, without a comparative characteristic, this feature is not useful in determining sex. Width of protarsomere 1(♀: 0.199 ± 0.00027 mm; ♂: 0.195 ± 0.00050 mm) and the combined length of protarsomere 2–4 (♀: 0.61 ± 0.00 mm; ♂: 0.61 ± 0.00 mm) varied little between sexes. However, both the ratio of protarsomere 1 length: width (♀: 2.69 ± 0.0045; ♂: 1.88 ± 0.0059; *F* = 11971.93, df = 1, 198, *p* < 0.0001) and the ratio of the combined length of protarsomeres 2–4 to the length of protarsomere 1 (♀: 1.14 ± 0.0015; ♂: 1.67 ± 0.0033; *F* = 21027.18, df = 1, 198, *p* < 0.0001) differed significantly by sex and there was complete separation (no overlap) for males and females (Figure 8). The form of the apex of the terminal sternite provided clear separation of the sexes. The terminal sternite is always rounded posteriorly in females (Figure 6) and always quadrate in males (Figure 5) based on our observations of 100 males and 100 females. Although the apices of the male protibial teeth can become more rounded through wear over time, the more acute (males) versus the less acute (females) distinction was reliable in over 90% of the cases for all three protibial teeth, with the apical tooth in males being the most consistently acute of the three teeth (Figures 3, 4, 9).

## Discussion

Three reliable characteristics that allow nondestructive determination of males and females of *A. sinicus* were found: 1) the form of the terminal sternite (Figures 5, 6); 2) the length to width ratio of protarsomere 1 (Figures 3, 4, 8), and; 3) the ratio of the combined length of protarsomeres 2–4: length of protarsomere 1 (Figures 3, 4, 8). These characteristics are most easily discerned in the laboratory with chilled specimens and with the aid of a microscope. It is also possible to discern sex differences in the field with a hand lens. For field application, it is easier to see the leg characters than the terminal sternite character.

In *A. sinicus* males, the apex of the terminal sternite is quadrate (Figure 5), whereas the apex of the female terminal sternite is rounded posteriorly (Figure 6). Cream-colored setae at the apex of the sternites may obscure this region slightly, but the characteristics are still discernable. This distinction was valid in all specimens examined. This characteristic is useful in many groups of Scarabaeidae, and it is broadly applicable within the genus *Adoretus* and other members of the tribe Adoretini.

The relative dimensions of the protarsomeres (Figures 3, 4, 8) in ventral view are also reliable for separating the sexes. This difference can be observed by comparing the combined length of protarsomeres 2 to 4 to the length of protarsomere 1, or by comparing the relative length and width of protarsomere 1. In *A. sinicus* males, the combined length of protarsomeres 2 – 4 is over 1.5 times the length of protarsomere 1 (Figures 3, 8), whereas in females, the combined length of protarsomeres 2 to 4 is less than 1.2 times the length of protarsomere 1 (Figures 4, 8). The relative proportions of protarsomere 1 is also a reliable character for sex determination. In *A. sinicus* males, the length averages less than 2 times the width. In females, the length averages greater than 2.5 times the width. Protarsomere characteristics must all be viewed ventrally; examination in dorsal view may lead to incorrect assessment of the length of tarsomeres. Protarsomere characteristics may be useful in sex determination of some other species of *Adoretus*, but are not broadly applicable within the entire genus. Characteristics of the protarsomeres have some applicability to some other scarabaeids (e.g., Japanese beetle, *Popillia japonica*, USDA-APHIS 2004) but must be assessed on a species by species basis.

Less reliable methods for separating the sexes involved relative characters that required comparison between known males and females as well as characteristics that were subject to abrasion and wearing. One of the characteristics was the difference in the form of the protibial teeth (acute in the males versus rounded in the females). Even restricting our observations to adults “aged” at least two weeks, the protibial teeth of males were more acute than those of females in over 90% of specimens examined. However, *A. sinicus* adults use the protibia for digging in the soil, and the teeth are subject to wear. Thus, this is an unreliable characteristic for sex determination and does not provide 100% accuracy.

For the sexing that we have done in the laboratory, we typically look first at the relative dimensions of protarsomere 1, and then confirm sexing based on this character with subsequent observations of the terminal sternite and the protibial teeth. As one develops familiarity with the differences, one can recognize the differences in the relative dimensions of protarsomere 1 without having to make any actual measurements. It's helpful to have more than one characteristic to use to reduce chance of incorrect decision-making based on assessment of only one character.

Other characters useful for determination of the sexes in other Rutelinae are, for example, the form of the abdomen (males with sternites concave or planar; females with sternites weakly convex), but this is not useful in the *Adoretus* species that we examined. In addition, characters that are useful in other *Adoretus* species are not reliable for use in *A. sinicus.* Those characters include: (1) the presence (female) or absence (male) of a longitudinal furrow on the disc of the pygidium; (2) the sternites appearing slightly convex (females) versus slightly concave (males); and (3) the 5^th^ protarsomere similar in width to other protarsomeres and armed with less developed internomedial teeth or lacking internomedial teeth (female) versus the 5^th^ protarsomere being generally wider and better developed than other protarsomeres and armed with well developed internomedial teeth (males) (e.g., Figures 12, 13).

This latter characteristic is apparent in *A. tenuimaculatus. Adoretus sinicus* was originally referred to as *A. tenuimaculatus* in Hawaii. Some literature and databases still confuse these species. The comparisons between these species are useful to illustrate differences (and similarities) between *Adoretus* species. Comparisons are more readily made between these two species because *A. tenuimaculatus* is one of the most commonly encountered *Adoretus* in collections, thus it is a good species for comparison due to accessibility of specimens. In males of *A. tenuimaculatus*, the fifth protarsomere is slightly thickened and armed with an internomedial tooth (Figure 13), whereas in females the fifth protarsomere is gracile and only slightly developed internomedially. This character was not useful, however, in separating males and females of *A. sinicus.* Other characters that may be useful in sex determination of other *Adoretus* species include the form of the pygidium (short and oblique in the female; longer and more convex in the male), size of the eyes (larger in the male than in the female), the length of the antennal club (longer in the male than in the female), and form of the pro- and mesotarsal claws (unequally split in the male; equally split in the female). These additional characteristics should not be applied generally to all species of *Adoretus.* However, they may provide some utility for research on other species.

**Figures 10–13.**
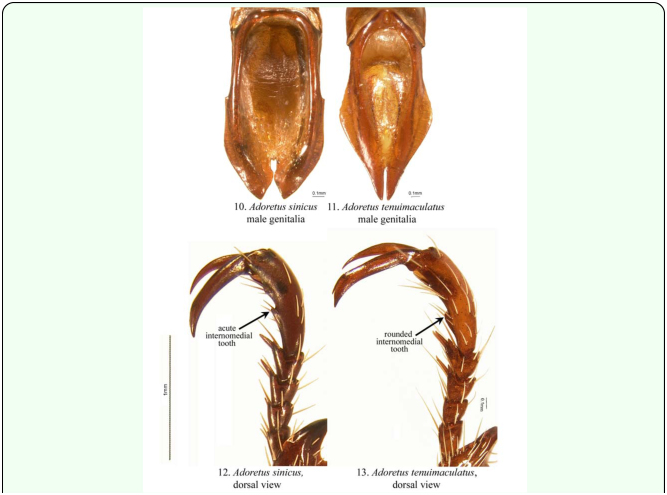
Characteristic differences between *A. sinicus* and *A. tenuimaculatus.* 10) Male parameres of *A. sinicus.* 11) Male parameres of *A. tenuimaculatus.* 12–13) Protarsus, dorsal view: 12) *A. sinicus*, male (with acute internomedial tooth on fifth protarsomere). 13) *A. tenuimaculatus*, male (with rounded internomedial tooth on fifth protarsomere). High quality figures are available online.

The genus *Adoretus* includes approximately 460 species (Krajcik 2007), and its taxonomy, biology, and evolutionary history are poorly known. *Adoretus sinicus* is not the only *Adoretus* species of biosecurity concern. The genus *Adoretus* includes several pests and invasive species in addition to the Chinese rose beetle (Table 1): *A. bicolor* Brenske, *A. caliginosus* Burmeister, *A compressus* (Weber), *A. hirsutus* Ohaus, *A. ranunculus* Burmeister, *A. tenuimaculatus* (Figure 11), and *A. versutus* Harold. Species in the genus are distributed in the Palearctic, Afrotropical, Indomalaysian, Australasian, and Oceanic biogeographic regions. Based on limited biological information, adults are herbivore generalists and are known to feed on a wide variety of plants. Larvae are associated with roots and, depending on the species, feed on living or non-living plant tissues. Eggs and larvae may be easily transported with cultivated plants in soil or roots (Ohaus 1935) which, combined with the generally broad host ranges, are the basis for the biosecurity concern for this group in relation to movement of potted plants across geographic regions. This biosecurity concern makes improved taxonomic knowledge of the group essential. Adult species in the genus are externally similar (about 10.0 mm in length, brownish with cream-colored scales) making identification of Adoretini problematic. Other than scattered and older regional works (*e.g*, Péringuey 1902: Arrow 1917; Baraud 1985), there are few resources for identification of species. Form of the male genitalia is probably the best method of identification (e.g., *A. sinicus* versus *A. tenuimaculatus* [Figures 10, 11]), and this necessitates dissection of the male genitalia. Molecular tools, however, are being developed to aid in the identification of adults and larvae. DNA in larval-adult species associations within scarab beetle communities from Nepal have been examined (Ahrens et al. 2007). Based on about 1600 base pairs of mitochondrial COX1 and RRNL and 700 base pairs of nuclear 28S rRNA, larval specimens could be associated with 19 identified, known adult species between 86.1% and 92.7% of the time (Ahrens et al. 2007). Nine morphotypes in the sample were members of the genus *Adoretus*, but only two morphotypes (22%) could be identified to species. In comparison, all four morphotypes within the genus *Maladera*(Coleoptera: Scarabaeidae: Melolonthinae; another scarab genus that includes pests) were identified to species. Clearly, additional systematics research is needed within the genus *Adoretus*, a group that includes many economically important species.

**Table 1.**
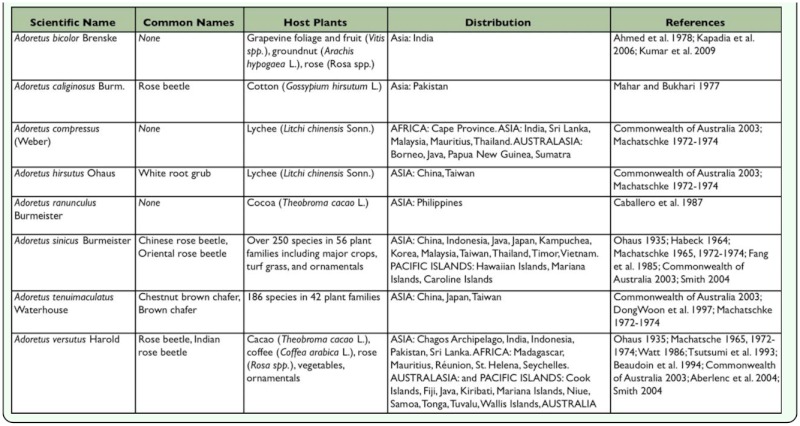
*Adoretus* species of biosecurity concern

The reliable, non-destructive means of *A. sinicus* sex determination reported here will be instrumental in research aimed at developing improved integrated pest management systems for *A. sinicus* as well as for other *Adoretus* species. Non-destructive means of sex determination of live specimens will facilitate progress in research that seeks to develop sex-dependent detection, monitoring and control methods that make use of pheromones, mating, or reproductive parameters. Such tools are of critical importance for managing existing pest populations as well as for countering new invasions of *Adoretus* species that are likely to occur given the magnitude of transport of agricultural goods across geographic regions.
